# Clustering-Based Plane Segmentation Neural Network for Urban Scene Modeling

**DOI:** 10.3390/s21248382

**Published:** 2021-12-15

**Authors:** Hongjae Lee, Jiyoung Jung

**Affiliations:** 1 Department of Electronic Engineering, Kyung Hee University, Yongin-si 17104, Korea; jimmy9704@khu.ac.kr; 2 Department of Artificial Intelligence, University of Seoul, Seoul 02504, Korea

**Keywords:** point cloud plane extraction, 3D point clustering, 3D segmentation, urban mapping

## Abstract

Urban scene modeling is a challenging but essential task for various applications, such as 3D map generation, city digitization, and AR/VR/metaverse applications. To model man-made structures, such as roads and buildings, which are the major components in general urban scenes, we present a clustering-based plane segmentation neural network using 3D point clouds, called hybrid K-means plane segmentation (HKPS). The proposed method segments unorganized 3D point clouds into planes by training the neural network to estimate the appropriate number of planes in the point cloud based on hybrid K-means clustering. We consider both the Euclidean distance and cosine distance to cluster nearby points in the same direction for better plane segmentation results. Our network does not require any labeled information for training. We evaluated the proposed method using the Virtual KITTI dataset and showed that our method outperforms conventional methods in plane segmentation. Our code is publicly available.

## 1. Introduction

Large-scale 3D reconstruction has been one of the most popular research topics in the field of robotics and computer vision for decades. In recent years, researchers and companies have been actively utilizing various sensors to obtain large-scale 3D reconstruction results over a certain level of accuracy with less time and cost. In particular, range sensors, such as LiDARs, have become very popular in outdoor scene modeling, and many researchers and engineers in related fields are now interested in utilizing the obtained large point clouds.

In this study, we focus on urban scene modeling, which is a challenging but essential task for 3D map generation for autonomous cars, city digitization, and AR/VR/metaverse applications. To model the ground and buildings, which are the major components in general urban scenes, we present a clustering-based plane segmentation neural network using 3D point clouds.

Plane segmentation from a point cloud is considered a foundation system in various computer vision and robotics fields, including object detection, model reconstruction, and map compression. Although many effective methods [1,2,3] have been proposed to segment a point cloud into planes, these methods focus on organized point clouds, which are depth values in grid arrays. However, point clouds obtained from LiDARs are usually unorganized, and organized point clouds obtained from depth cameras can easily be expressed in an unorganized form as well. Random sample consensus (RANSAC) and region-growing-based algorithms have been widely used to extract planes from unorganized 3D point clouds.

**Figure 1:**
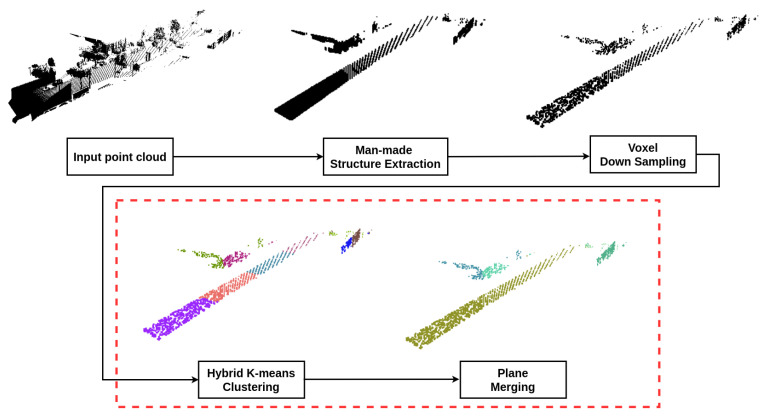
The plane segmentation procedure using the proposed system. We extract man-made structures from the unorganized input point cloud and voxelize the points using voxel down sampling as pre-processing. Then, the proposed hybrid K-means clustering method segments the point cloud into planes. Finally, the merging process combines the small planes that need to be merged.

**Figure 2:**
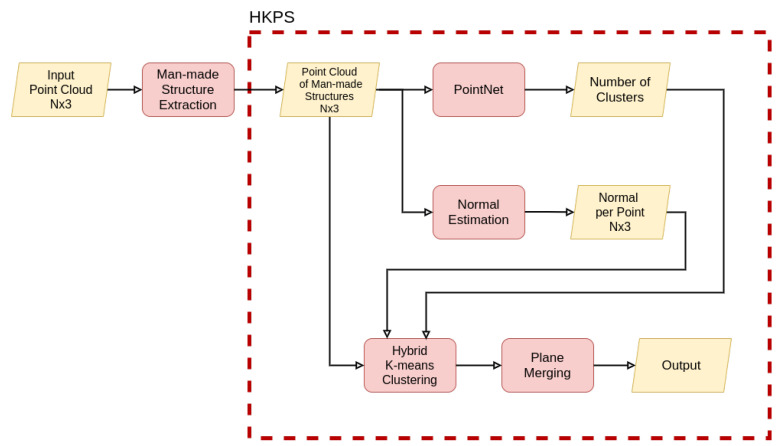
An overview of the proposed framework. PointNet [6] takes the input point cloud of man-made structure and outputs the number of clusters. We estimate the normal of each point, and then perform hybrid K-means clustering for plane segmentation (Section 3.1 and Section 3.2). At the end of the network architecture, the plane merging process optimizes the number of planes (Section 3.3).

We propose a novel approach to segment unorganized 3D point clouds into planes, called hybrid K-means plane segmentation (HKPS). The whole plane segmentation procedure using the proposed system is shown in Figure 1, and the detailed plane segmentation process of HKPS is shown in Figure 2. We extracted man-made structures from the unorganized input point cloud and voxelized the point using voxel downsampling as pre-processing. The proposed HKPS involves two steps: hybrid K-means clustering and plane merging. Hybrid K-means clustering performs clustering based on K-means++ [4], which is a K-means [5]-based clustering algorithm that has the advantage of careful seeding. K-means++ requires a parameter *K*, which is the number of clusters. We used PointNet [6] to estimate parameter *K* from the point cloud. PointNet is an unsupervised learning model that uses cosine distance for training. The final plane merging step combines the small planes that need to be merged. Our code is publicly available at [7].

## 2. Related Work

Semantic segmentation in 2D images using convolutional neural network (CNN) has been extensively studied in several decades [8,9,10]. Semantic segmentation in 3D point clouds using CNN is a more challenging task in many aspects, and researchers have focused on the related problems [6,11,12], including the recent studies on fast and efficient 3D segmentation for urban scene analysis [13,14,15]. For the task of segmentation in point clouds, many previous works [16,17] used PointNet [6]. These previous approaches require the ground truth for training the neural network, which is usually difficult to obtain. Labeling 3D points for plane segmentation is a laborious task owing to the noise and ambiguity in point clouds. More recent methods that do not require ground truth are usually based on the Hough transform, RANSAC, and region growing.

### 2.1. Hough Transform

Hough transform [18] is a feature extraction technique used to find objects with a certain class of shapes by a voting procedure. This voting procedure is carried out in a parameter space, from which object candidates are obtained as local maxima. The classical Hough transform is used to detect lines in binary images. The key idea behind the Hough transform is to change the coordinate system from image to Hough space. If the coordinate system is moved to the Hough space, lines passing through the points can be displayed as intersection points. Vosselman et al. [19] presented a plane extraction method from 3D point clouds based on the Hough transform and proposed a 3D Hough transform using normal vectors to enhance the speed and reliability of the traditional Hough transform. Limberger and Oliveira [20] proposed a plane extraction algorithm for point clouds by extending the kernel-based Hough transform [21]. The method creates a cluster using an octree and principal component analysis (PCA) and then votes for each cluster using a Gaussian kernel to extract planes. These methods are computationally fast and effective but tend to be sensitive to outliers.

### 2.2. RANSAC

Random sample consensus (RANSAC) was first presented by Fischler and Bolles [22]. RANSAC randomly selects some sample data and finds the model parameters to represent the sample data. In the case of finding planes to fit the sample points, the model parameters are the parameters in the plane equation. To find the best parameters, the method counts the number of inliers for the selected model, repeats the process several times, and finally selects the model with the largest number of inliers as the final result. Typically, to extract planes from 3D point clouds, we run RANSAC sequentially until no more planes can be found. RANSAC-based plane extraction algorithms are computationally more expensive but usually more robust to noise than Hough transform-based algorithms. However, these methods tend to combine adjacent planes into the same plane, specifically when the size of the adjacent plane is small.

Gotardo et al. [23] used a robust estimator to avoid premature convergence, which resulted in the preservation of small regions and edge locations, and accelerated the optimization process by a genetic algorithm. Schnabel et al. [24] predicted a model using only nearby peripheral points with an octree. The method detects planes, spheres, cylinders, cones, and tori and shows improved speed and scalability. Point sets with several million samples are robustly decomposed within less than a minute. Gallo et al. [25] proposed CC-RANSAC, which only considers the largest connected components of inliers to evaluate the fitness of a candidate plane. CC-RANSAC recovers the planar patches composing a typical step or ramp with higher accuracy than the traditional RANSAC algorithm. Despite recent advances, RANSAC based algorithms are still weak in distinguishing small clusters of points. They usually fail to produce reliable results in situations with two nearby patches of limited extent, where a single plane crossing through the two patches may contain more inliers than the “correct” models.

### 2.3. Region Growing

The region growing algorithm works by randomly selecting a starting point called a seed from the point cloud and then growing the region by adding points that satisfy a set of criteria. PCA is generally used to calculate the normal and curvature required for regional growth. Nurunnabi et al. [26] proposed a minimum covariance determinant (MCD)-based robust PCA to solve the outlier sensitivity problem of PCA. Vo et al. [27] created an octree and generated planar patches using PCA at each node of the octree. Their method measures the planarity of planar patches using the mean square distance, and subdivides the patches recursively with a threshold. After extracting planar patches, planes and points are merged using the difference between the normal angle of each plane and its distance to the point. Araújo and Oliveira [28] presented a plane extraction algorithm that does not require parameter tuning. Their method is similar to that of Vo et al., but it creates a planar patch in an octree by a bottom-up approach, whereby it keeps subdividing until planes can be detected. Planarity tests are performed using angular differences at each step to avoid parameter tuning. Region growing methods tend to fail in extracting a plane composed of few points similar to RANSAC-based methods. They also show difficulties in dividing two adjacent planes with different directions at the edges.

### 2.4. Clustering

Clustering algorithms can be classified as hierarchical or non-hierarchical. Most previous clustering-based plane segmentation algorithms are based on hierarchical clustering. Feng et al. [1] presented a plane extraction algorithm using hierarchical clustering. After extracting planar patches from the point cloud using PCA, the plane extraction algorithm constructs a graph to represent the patches and their relationship with each other. Then, agglomerative hierarchical clustering is performed using the mean-squared orthogonal point-to-plane fitting error. Finally, the roughly extracted planes are fine-tuned using the region growth in pixels. Schaefer et al. [3] utilized the maximum likelihood estimation (MLE) for agglomerative hierarchical clustering. When clustering, a plane is always extended in the direction that decreases the measurement likelihood of the scan. However, these methods can only be applied to organized point clouds.

Non-hierarchical clustering can be divided into density-based and center-based methods. Ester et al. [29] proposed a density-based clustering algorithm, named DBSCAN, based on the assumption that similar data are closely distributed to each other. Continuously dense regions are defined as clusters. DBSCAN performs well when dense and non-dense areas are easily distinguished. K-means clustering [5], a center-based method, performs clustering with *K* clusters while moving the centroids in a direction that minimizes the distance between the centroid and points in each cluster. However, it has a disadvantage in that it requires an appropriate parameter *K* to cluster the point cloud. To solve the problem of a random selection of initial centroids, Arthur and Vassilvitskii [4] proposed an improved version of the initial centroid selection and named the method K-means++. The improved method uses distance proportional probabilities to select a new centroid as far as possible from other centroids.

In this study, we propose a plane segmentation algorithm for unorganized point clouds based on the K-means++ non-hierarchical clustering algorithm. While the conventional K-means++ clustering algorithm only measures the Euclidean distance for clustering, the proposed hybrid K-means clustering method considers both the Euclidean distance and cosine distance for plane segmentation. Therefore, our method better distinguishes small plane segments located far from the sensor or partly occluded by obstacles, which RANSAC and region growing-based methods tend to miss. The requirement of setting *K* is also resolved by training a neural network without any labeled data to estimate an appropriate number of clusters for the point cloud.

## 3. Hybrid K-Means Plane Segmentation Neural Network

In this section, we first describe how the proposed hybrid K-means clustering segments a point cloud into planes. Next, we explain how PointNet estimates the number of planes. Finally, we demonstrate how to merge planes extracted from hybrid K-means clustering that need to be combined into the same plane.

### 3.1. Hybrid K-Means Clustering

K-means++ clustering is one of the most popular algorithms for segmenting data into k clusters. Nevertheless, K-means++ clustering is not suitable for segmenting a point cloud into planes because K-means++ clustering only considers the Euclidean distance for clustering. Spherical K-means clustering [30] is suitable for clustering high-dimensional data using the cosine distance between vectors. Inspired by K-means++ and spherical K-means clustering methods, we propose a hybrid K-means clustering method that clusters data into planes using both Euclidean distance and cosine distance. When hybrid K-means clustering measures the correlation between points, positional difference uses Euclidean distance, and normal difference uses cosine distance. Therefore, distant planes were clustered using Euclidean distances, and the directions of the planes were clustered using cosine distances. Therefore, distant planes are clustered using Euclidean distances, and the direction of the planes are clustered using cosine distances.

To estimate the normals of each point, we used K-nearest neighbors with the K-d tree provided by Open3D [31] for the entire point cloud. Open3D is an open-source Python library that supports rapid development of software that deals with 3D data. The K-d tree is a space-partitioning data structure that structures points in a K-dimensional space. The K-d tree is a useful data structure for finding the nearest neighbors. We set the maximum neighbors as 30, and the search radius as 3. We define the centroid of the i-th cluster as $c_{i}$ and each point in the i-th cluster as $x_{j}\in S_{i}$ . The goal of the hybrid K-means clustering is to find $S_{i}$ that minimizes the overall variance *V*:

(1) $$V=\sum_{i=1}^{K}\sum_{x_{j}\in S_{i}}^{}d\left(x_{j},c_{i}\right),$$

The function $d(x_{j},c_{i})$ computes the difference between $x_{j}$ and $c_{i}$ .

The algorithm starts by setting the initial cluster centroids $c_{i}$ . K-means-based clustering algorithms have a disadvantage in that their performance varies greatly depending on how the initial value is selected. To overcome this disadvantage, we set the initial cluster centroids $c_{i}$ using K-means++, which has the advantage of careful seeding. After selecting the initial centroids, we allocate each point to the most similar cluster by using $d(x_{j},c_{i})$ , which is the difference between $x_{j}$ and $c_{i}$ . $d(x_{j},c_{i})$ is the sum of the Euclidean distance and cosine distance, as shown in Equation (2).

(2) $$d\left(x_{j},c_{i}\right)=E\left(x_{j},c_{i}\right)+\lambda C\left(x_{j},c_{i}\right)$$

(3) $$E\left(x_{j},c_{i}\right)=\sqrt{{}^{}{∥x_{j}-c_{j}∥}^{2}}$$

(4) $$C\left(x_{j},c_{i}\right)=1-\frac{x_{j}\cdot c_{j}}{\left|x_{j}\right|\left|c_{j}\right|}$$

$E(x_{j},c_{i})$ computes the Euclidean distance between positional differences and $C(x_{j},c_{i})$ computes the cosine distance between normal differences, as shown in Equations (3) and (4). λ is a parameter that determines the ratio between the Euclidean distance and the cosine distance. As λ grows, parallel planes are classified as the same plane, owing to the increased influence of the cosine distance. In contrast, if λ is too small, the point cloud cannot cluster as planes. For the clustering points into planes, we chose λ = 60. After allocating each point to the most similar cluster, we rearrange the centroid $c_{i}$ of cluster $S_{i}$ using the center of gravity of points in $S_{i}$ . Equation (5) shows how to rearrange the centroid $c_{i}$ .

(5) $$c_{i}=\frac{1}{\left|S_{i}\right|}\sum_{x_{j}\in S_{i}}^{}x_{j}$$

The proposed hybrid K-means clustering repeats the above two steps until the centroids converge.

### 3.2. Parameter Estimation

The proposed hybrid K-means clustering method has the disadvantage that *K*, the number of planes to be extracted, must be passed as an input parameter. We used PointNet to automatically determine parameter *K* from the point cloud. PointNet takes an unorganized point cloud represented in the format of $R^{N\times 3}$ as the input, where *N* is the number of points. PointNet is a widely used library in the field of computer vision for classification and segmentation by analyzing the characteristics of input point clouds. Our purpose is to train PointNet to infer parameter *K*, which is the number of planes. In general, a pre-processing process for labeling a dataset is essential for obtaining labeled point clouds. However, in this study, we propose an unsupervised training method using hybrid K-means clustering.

After hybrid K-means clustering, the input points are segmented into *K* planes. The average cosine distance $L_{k}$ between each plane’s centroid $c_{i}$ and their assigned *M* points $X=\left\{x_{1},x_{2},\cdots ,x_{M}|x_{j}\in S_{j}\right\}$ can be defined by

(6) $$L_{k}=\sum_{i=1}^{k}\frac{1}{M}\sum_{x_{j}\in S_{j}}^{M}C\left(x_{j},c_{i}\right).$$

**Figure 3:**
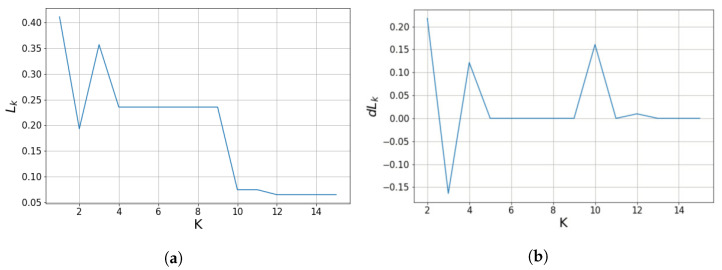
(**a**) Average cosine-distance value of a point cloud calculated by Equation (4) according to *K*, the number of planes. (**b**) Difference of average cosine-distance calculated by Equation (7). If *K* is sufficient to cluster point cloud into planes, we can see that the difference of average cosine-distance converges close to zero and no longer changes. By using difference of average cosine-distance, we can obtain the appropriate parameter *K*.

As *K* increases, $L_{k}$ decreases when the point cloud is divided into planes, as shown in Figure 3a. We use $dL_{k}$ , which is the difference of $L_{k}$ , which can be obtained as the difference between $L_{k-1}$ and $L_{k}$ .

(7) $$dL_{k}=L_{k-1}-L_{k}$$

If *K* is sufficient to cluster the input point cloud into planes, we can see that the difference in the average cosine distance converges close to zero and no longer changes, as shown in Figure 3b. Therefore, the smallest parameter *K*, in which $dL_{k}$ begins to converge close to zero is the appropriate number of planes for the hybrid K-means clustering method. We experimentally define that $dL_{k}$ converges close to zero when $\left|dL_{k}\right|<0.1$ is satisfied. Because of $L_{k}$ and $dL_{k}$ , as shown in Figure 3, the value of *K* for the with-noise-00 point cloud was automatically selected as 11. PointNet can be trained in an unsupervised manner using the *K* obtained by the above method. PointNet requires a parameter that sets the maximum limit of *K*, which we set to 15.

The parameter estimation for hybrid K-means clustering using PointNet has an advantage in terms of computational time. It takes a lot of time to train PointNet, but after training it is more time efficient than without using PointNet, which involves performing hybrid K-means up to the maximum limit of *K* each time to obtain the appropriate parameter *K*.

### 3.3. Plane Merging

In the previous sections, we clustered a point cloud into planes with an appropriate number of planes. Figure 1 illustrates the process of the proposed approach. The proposed hybrid K-means clustering sometimes oversegments input points into two or more planes that eventually need to be grouped into the same plane, owing to the Euclidean-distance term in Equation (3). Figure 1 shows that hybrid K-means clustering segments the road into four planes, even though it can be merged into a single plane. Therefore, we present a plane-merging algorithm that combines planes that should be merged into the same plane. To merge different planes, the following two conditions must be satisfied: angular similarity and distance limit. To find a plane $S_{j}$ that satisfies the condition of angular similarity with another plane $S_{i}$ , we compute the cosine distance between centroid $c_{i}$ of plane $S_{i}$ and centroid $c_{j}$ of plane $S_{j}$ . If the cosine distance is smaller than threshold σ , we determine that the angular similarity condition is satisfied. We create a new set *I* that consists of planes satisfying the condition of angular similarity with plane $S_{i}$ , as shown in Equation (8).

(8) $$I=\left\{S_{j}\in S|C\left(c_{i},c_{j}\right)<\sigma \right\}$$

If we only consider angular similarity, different planes facing in the same direction apart from each other can be merged into the same plane. To avoid such situations, we also consider the distance limit condition. If the closest points in planes $S_{j}$ and $S_{i}$ are closer than threshold ω , we consider that the planes satisfy the distance limit condition. Such planes are collected from *I* to form set *H*, as described in Equation (9).

(9) $$H=\left\{S_{j}\in I|\left|SE(S_{i},S_{j})\right|>1\right\}$$

(10) $$SE\left(S_{i},S_{j}\right)=\left\{x_{i}\in S_{i}|E\left(x_{i},x_{j}\right)<\omega \right\}$$

$\left|\cdot \right|$ represents the cardinality of the set. $SE(S_{i},S_{j})$ is the set of points belonging to planes $S_{i}$ , where the Euclidean distance between points is closer than threshold ω , as in Equation (10). The members of *H* merge with plane $S_{i}$ to form a new merged plane $S_{m}$ . The above process is repeated until there are no additional planes to merge. We set σ=0.1 and ω=4 .

## 4. Results

To evaluate the proposed hybrid K-means plane segmentation (HKPS) method, we used the photorealistic synthetic Virtual KITTI Dataset [32], which closely mimics the real-world KITTI dataset [33]. The Virtual KITTI dataset was labeled with 13 different labels. The dataset obtains a semantically annotated 3D point cloud by projecting a given 2D depth into a 3D space. We extracted man-made structures, such as roads and buildings, from the Virtual KITTI dataset to evaluate the plane segmentation performance. This is because these man-made structures can be expressed in planes, which are effective in composing an urban scene model. The Virtual KITTI dataset consisted of unorganized point clouds.

For comparison evaluation, we tested the following most widely used plane segmentation techniques: RANSAC, region growing, and K-means clustering-based techniques. For RANSAC, we used the pyRANSAC-3D library [34] to fit the planes in a point cloud. For region growing, we used the latest technology, RSPD [28]. RSPD had the advantage of extracting planes robustly against noise, and it exhibited better performance in various indoor environments than the existing plane segmentation techniques. For K-means clustering, we used K-means++, which is most similar to our technique among the previous methods. In the comparison experiment with K-means++, PointNet was trained using K-means++ in the same way as HKPS to determine the number of clusters.

PyTorch was used to implement the proposed HKPS. The training was performed on a single NVIDIA 2080Ti GPU. We used the Open3D library [28] to find the normal vectors in the point cloud and implement voxel downsampling.

### 4.1. Voxel Down Sampling

**Figure 4:**
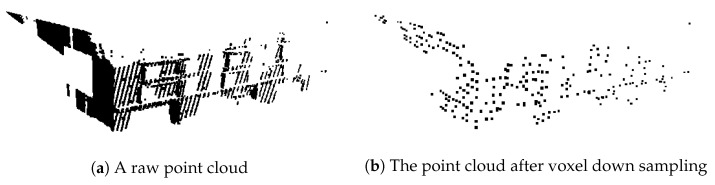
The same point cloud (**a**) before and (**b**) after voxel down sampling. The density of the point cloud becomes uniform and noise is removed.

The point cloud is partially composed of different densities owing to the distance from the sensor, angle, and obstacles, as shown in Figure 4a Because hybrid K-means clustering rearranges the centroids using the center of gravity of each cluster, the centroids tend to be rearranged in the direction with a greater density of the points. To solve this problem, we perform voxel downsampling, and the result is shown in Figure 4b.

A voxel downsample filter combines a three-dimensional voxel grid on a point cloud. The points inside each voxel were downsampled to the center of each voxel. The process of voxel downsampling reduces the number of points to align with a uniform density. This process not only benefits from balanced centroid rearrangement for hybrid K-means clustering but also reduces noise and the number of input points to PointNet. Reducing the number of points enables efficient use of memory. After voxel downsampling, we extracted 1600 points from each point cloud for PointNet training.

### 4.2. Performance Evaluation

Two types of datasets are used to compare the plane segmentation performance of our proposed method, HKPS, with previous works: *with-noise* and *without-noise* datasets. The *with-noise* dataset is the Virtual KITTI dataset after voxel downsampling with uniform density. For the *without-noise* dataset, we deleted small clusters of points, such as parts of trees, vehicles, and unknown objects, from the *with-noise* dataset. PointNet was trained with 70 scenes from the *with-noise* dataset, and the performance evaluation experiment was performed with 20 scenes from each of the *with-noise* and *without-noise* datasets.

We used the performance metrics proposed by Hoover et al. [35] for quantitative comparison with previous works, and the results are shown in Table 1 and Table 2. When plane $S_{i}$ of the ground truth and plane $S_{j}$ segmented by the evaluation method overlap threshold T% or more, we classify the plane as **correct detection** and use threshold T=80 . **Over segmentation** means that a plane is segmented with a greater number than it should be, and **under segmentation** means that it is segmented with a smaller number of planes. **Missed** means that plane $S_{i}$ of ground truth does not participate in any correct detection, over segmentation, or under segmentation. **Noise** means that plane $S_{j}$ segmented by evaluation method does not participate in any correct detection, over segmentation, or under segmentation.

To increase the objective validity of the results, we propose an average cosine distance (ACD) as an additional performance evaluation metric.

(11) $$ACD=\sum_{i=1}^{N}\frac{1}{M}\sum_{x_{j}\in S_{j}}^{M}C\left(x_{j},c_{i}\right).$$

The average cosine distance represents the angular difference between the normal of each point and the centroid normal of the plane. *N* is the number of planes extracted from this method.

**Figure 5:**
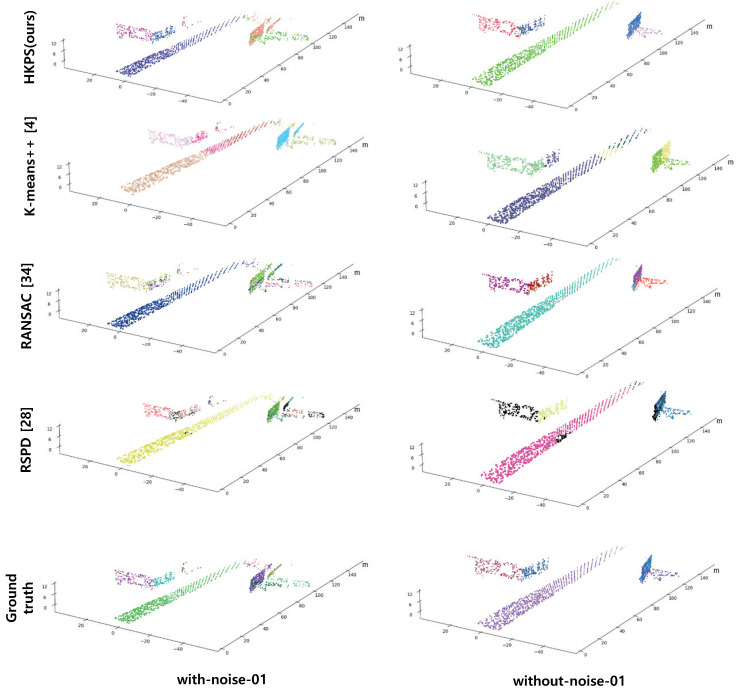
Performance comparison results using scene 01 from *with-noise* Virtual KITTI Dataset and *without-noise* Virtual KITTI Dataset. Different planes are displayed with different colors. The outliers of RANSAC and RSPD are colored in black.

**Figure 6:**
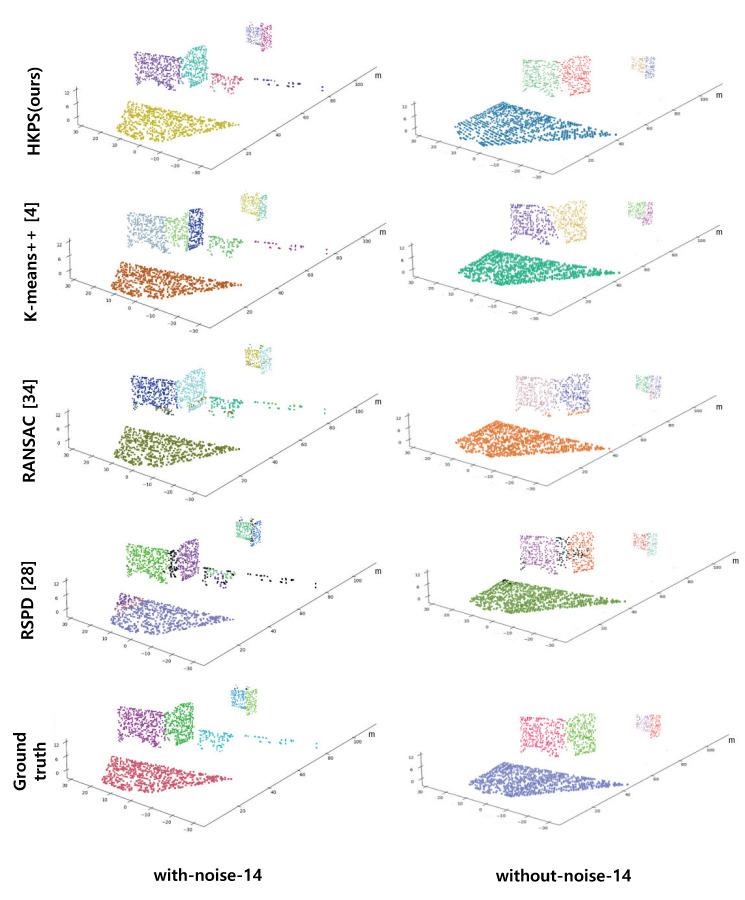
Performance comparison results using scene 14 from *with-noise* Virtual KITTI Dataset and *without-noise* Virtual KITTI Dataset. Different planes are displayed with different colors. The outliers of RANSAC and RSPD are colored in black.

It is necessary to define the ground truth suitable for plane segmentation to evaluate the proposed plane segmentation technique. Because the ground truth was not available for the Virtual KITTI dataset, we created it by labeling 3D point clouds using RGB 2D images provided by the dataset. The ground truth is shown in the last lines of Figure 5 and Figure 6.

K-means++ clustering achieved the lowest correct detection in both the *with-noise* and *without-noise* datasets, and it achieved the largest average cosine distance in the *without-noise* dataset and the second largest in the *with-noise* dataset. As shown in Figure 5, K-means++ fails to segment the corners of buildings and falsely segments buildings and roads into the same cluster if the distance between the building and the road is small. This is because K-means++ clustering only considers the Euclidean distance when segmenting clusters; therefore, it is not suitable for clustering point clouds into planes.

RANSAC showed large values of missed and noise in both datasets, and the average cosine distance was the largest in the *with-noise* dataset, which was significantly higher than that in the *without-noise* dataset. This is because the points in the *with-noise* dataset are widely distributed in the horizontal direction of the surface owing to noise. As shown in the *with-noise* scene in Figure 5, RANSAC creates planes perpendicular to the buildings because the method segments planes without considering their normals. The performance depends greatly on the initial seed selection. Because of these disadvantages, RANSAC cannot extract small planes, and the planes that are scanned only partially by the sensor are estimated as noise.

RSPD has the second highest correct detection in the *without-noise* dataset, but it drops significantly in the *with-noise* dataset. Despite the low value of correct detection, RSPD has the smallest average cosine distance compared to the other methods. RSPD determines outliers using angle difference to extract planes from various datasets without adjusting parameters; however, the allowable angle difference is tightly set. As shown in Figure 6, the planes composed of a few points in the *with-noise* dataset and the corners that are difficult to be segmented into planes using angle differences were classified as outliers.

HKPS has the highest correct detection in both datasets, and its average cosine distance is the second best. This means that the proposed HKPS segments the point cloud into appropriate planes. The values of missed and noise are significantly lower than those of other methods, as the clustering-based method of HKPS can segment the planes with a small number of points. It is shown that HKPS is suitable for segmenting planes in urban scenes, where many parts of planes are obscured by obstacles, such as vehicles and trees.

### 4.3. Scalability

**Figure 7:**
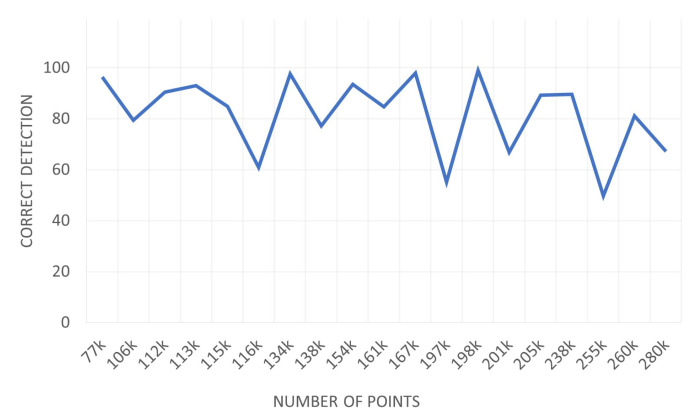
Correct detection metric difference according to the number of points. It can be seen that there is not much change in performance depending on the number of points.

We measured the performance change with the number of points in the input point cloud. Figure 7 shows a graph of the correct detection change according to the change in the number of input points. There may be more points in the urban scene, but we measured performance using 70,000 to 200,000 points.

The number of input points was the number before voxel downsampling, and 1600 points were extracted for training PointNet after voxel downsampling. As the number of points increases, it does not appear to be a significant drop in performance. This proves that our method is suitable for plane segmentation in an urban scene composed of a large number of points.

## 5. Conclusions

We presented an effective neural network for segmenting an urban scene point cloud into planes. Our plane segmentation method has two major differences compared to the previous methods. First, we combine two types of coordinate differences: Euclidean and cosine differences. Our experiments demonstrate that combining the two coordinate differences translates into superior accuracy plane clustering results. The proposed method segments the planes composed of a small number of points that are far from the sensor or partly obscured by obstacles. Second, we use PointNet as a method to automatically determine the number of planes in the point cloud. Previous K-means clustering-based methods applied various methods to find the appropriate number of clusters K. In addition, to find an appropriate K, it was necessary to perform clustering several times while changing K. However, the proposed method uses PointNet to estimate the number of clusters so that the plane can be segmented by performing clustering only once. Because of this, parameter estimation using PointNet has an advantage in terms of computational time.

Owing to the effective results, we suggest several extensions of the proposed plane segmentation method, HKPS. We plan to extend PointNet to enable parameter estimation that is suitable for various datasets. In addition, we are working on extending HKPS to a network that can extract other primitive types, such as spheres and cones.

## Figures and Tables

**Table 1:** Average result of *without-noise* Virtual KITTI Dataset. Best results are highlighted in bold.

Method	Correct Detection [%]	Under- Segmentation [%]	Over- Segmentation [%]	Missed [%]	Noise [%]	Avg. Cosine Distance
HKPS (ours)	**84.26**	3.18	11.31	**0.19**	**0.1**	0.0337
K-means++ [4]	70.56	0.76	17.27	9.41	9.40	0.0820
RANSAC [34]	72.36	**0.06**	6.97	13.28	13.69	0.0606
RSPD [28]	72.54	12.35	**0.07**	10.05	14.78	**0.0182**

**Table 2:** Average result of *with-noise* Virtual KITTI Dataset. Best results are highlighted in bold.

Method	Correct Detection [%]	Under- Segmentation [%]	Over- Segmentation [%]	Missed [%]	Noise [%]	Avg. Cosine Distance
HKPS (ours)	**83.80**	**1.46**	9.50	**2.76**	**2.10**	0.0315
K-means++ [4]	51.87	14.70	17.93	11.68	10.76	0.0916
RANSAC [34]	70.93	2.42	4.14	18.30	16.44	0.1284
RSPD [28]	65.96	9.94	**1.21**	17.11	18.69	**0.0106**

## Data Availability

The data presented in this study are openly available at reference number [32]. Our code is openly available at reference number [7].
